# Current Status and Perspectives Regarding the Treatment of Osteosarcoma: Chemotherapy

**DOI:** 10.2174/157488708785700267

**Published:** 2008-09

**Authors:** Akio Sakamoto, Yukihide Iwamoto

**Affiliations:** Department of Orthopaedic Surgery, Graduate School of Medical Sciences, Kyushu University, Japan

**Keywords:** Osteosarcoma, chemotherapy.

## Abstract

Osteosarcoma is the most common primary bone tumor in childhood and adolescence. The use of combination chemotherapy and surgery enables long-term survival in approximately 60-70% of cases. However, the necessity for surgery, the poor prognosis of patients with metastatic or recurrent disease (long-term survival in only about 20% of cases), and the lack of establishment of second-line chemotherapy suggest that improvements in chemotherapy are desperately needed. Currently, in an effort to extend the protocol with the chemotherapy drugs that already exist, high-dose chemotherapy with/without autologous peripheral blood stem cell transplantation, and tumor-targeted drug delivery systems are under investigation. Future drug developments will no doubt lie in the direction of immunotherapy and anti-angiogenic therapy, as well as the use of cytotoxic drugs. Identifying the genes and signal transduction pathways responsible for the development of osteosarcoma or for the occurrence of malignancy in cases of osteosarcoma will undoubtedly lead to the identification of pathway-specific agents, or possible gene therapy. Furthermore, as increased light is shed on the character of osetoblastic differentiation in osteosarcoma, this will certainly give rise to new treatments utilizing differentiation therapy. This article reviews the current status and perspectives regarding the treatment of osteosarcoma in terms of chemotherapy.

## INTRODUCTION

Osteosarcoma is the most common primary bone tumor in childhood and adolescence, and in about 80% of cases it occurs in the long bones of the limbs. In the other 20% of cases, it occurs in the axial skeleton and pelvis [1]. Commonly affected bones, in descending order, are the femur (40%), the tibia (20%) and humerus (10%) [2]. Osteosarcoma occurs primarily in the metaphysis of long bones around the knee region of the distal femur or proximal tibia. Osteosarcoma is highly aggressive and it metastasizes primarily to the lung [3]. The median age of an osteosarcoma patient is 16 years, with a male predominance (male/female ratio; 1.6:1). The occurrence of osteosarcoma seems to be associated with a spurt in growth [4]. The peak incidence among female patients has been reported to be a little earlier than that among male patients, probably because of the earlier onset of growth spurt at the growth plate [5, 6]. Histologically, osteosarcoma is characterized by a proliferation of atypical spindle cells. Malignant osteoid formation is diagnostic for osteosarcoma. Conventional osteosarcoma is classified into osteoblastic, chondroblastic and fibroblastic, according to its predominant features [7].

Genetic alterations have been reported in osteosarcoma. In particular, the tumor-suppressor pathways of p53 and Rb (retinoblastoma tumor-suppressor gene) are thought to be involved in the pathogenesis of osteosarcoma [8-11]. The majority of patients with osteosarcoma have neither a familial background nor a history of radiation exposure. Li-Fraumeni syndrome (germline deletion in p53) and familial retinoblastoma (germline mutations of Rb) are known to be risk factors in the development of osteosarcoma [10, 12-15]. Even osteosarcoma without such a background has alterations in both the p53 and Rb pathways in the majority of cases [7]. p53 is a tumor-suppressor gene and its product plays an important role in the cellular response to DNA damage [16]. Rb product has a suppressive effect on the cell cycle. p53 and Rb are thought to be involved in osteosarcoma oncogenesis [17-19]. Unbalanced karyotypes and other abnormal cellular signaling pathways have also been reported in osteosarcoma cells. Among possible cytogenetic alterations, gains in chromosome 1, loss of chromosome 9 and loss of heterozygosity on several chromosomes are consistently reported [7].

Mouse double minute 2 (MDM2) and cyclin-dependent kinase 4 (CDK4) genes have been reported to be amplified or overexpressed in osteosarcoma, and these genes are thought to be involved in the pathogenesis [20, 21]. The MDM2 gene product binds and inactivates p53 protein, whereas the CDK4 gene product is a cyclin-dependent kinase and possibly inactivates Rb function. c-fos is a transcriptional factor and controls cell-cycle progression. c-fos plays an important role in osteoblast and chondrocyte differentiation. Amplification or overexpression of the c-fos gene has been reported in osteosarcoma cells [22]. 

## CURRENT CHEMOTHERAPY

**Fig.(1):**
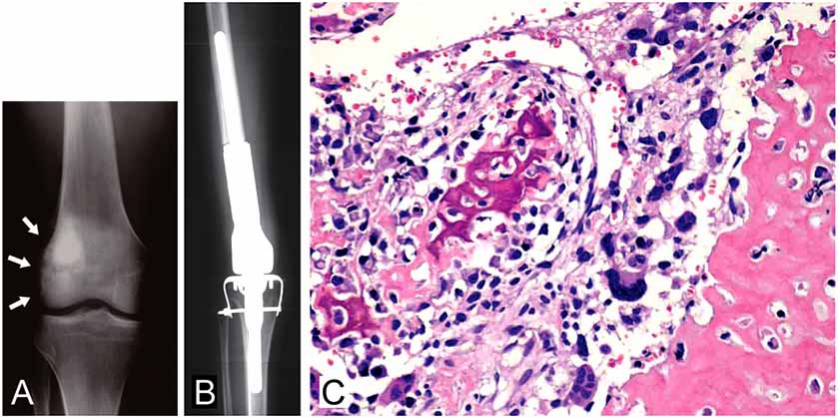
Osteoblastic osteosarcoma. Plain radiograph of a 19-year-old male clearly shows an irregular osteoblastic feature in the medullary region of the distal femur with cortical irregularity over the lateral cortex (arrows) (A). After a three-drug chemotherapy regimen made up of cispatin, doxorubicin and methotrexate, wide resection was carried out, followed by reconstruction using a Kotz prosthesis (HMRS: Howmedica Modular Reconstruction System) (B). Histological section reveals osteoblastic osteosarcoma clearly demonstrating osteoblastic features, comprising irregular immature bone deposition by anaplastic tumor cells (C).

Until the 1970s, osteosarcoma was treated by amputation in most cases, or else by radiotherapy. In spite of local control, most osteosarcoma patients died within a short period because of lung metastasis. The results of surgery alone as a treatment of osteosarcoma have not been satisfactory. The 5-year disease-free survival rate after treatment by surgery alone has been reported to be only 12% [3]. Neoadjuvant (preoperative) chemotherapy was introduced in 1978 [23]. The purposes of neoadjuvant chemotherapy are the destruction of primary tumor cells and the eradication of micrometastasis. Doxorubicin and methotrexate have been applied successfully as chemotherapy drugs for the treatment of osteosarcoma [24-27]. In the course of a study into osteosarcoma chemotherapy, vincristine, bleomycin and dactinomycin have all been proven to be ineffective [28, 29]. Subsequently, the addition of cisplatin and ifosfamide to doxorubicin and methotrexate has been able to improve clinical results significantly [30, 31]. The current standard protocol of a three-drug chemotherapy regimen using cispatin, doxorubicin and high-dose methotrexate provides about 70% long-term disease-free survival for osteosarcoma patients without metastasis (Fig. **1**) [7].

The amount of tumor necrosis following preoperative chemotherapy has been known to be a reliable prognostic factor, enabling the effectiveness of the chemotherapy treatment to be accurately determined [32, 33]. Tumor necrosis is usually assessed as follows: Grade I: no necrosis; Grade II: necrosis of between 50 and 95%; Grade III: necrosis greater than 95 but less than 100%; and Grade IV: total necrosis; 100% [23]. Patients with tumor necrosis in excess of (≥) 90% are classified as good responders to chemotherapy, whereas those whose tumor necrosis is less than (<) 90% are classified as poor responders.

### Therapy for Patients with Metastasized Osteosarcoma

Unfavorable prognosis is associated with tumor location, the number and location of the metastases and lack of adequate surgical resection. In particular, the prognosis of osteosarcoma patients with metastasis is still poor, with only 20% achieving a 5-year survival rate [1]. Treatment for patients with relapsed osteosarcoma following standard chemotherapy or for patients with tumors that are unresponsive to standard chemotherapy remains challenging. A second-line chemotherapy regimen for use when applied standard chemotherapy fails, has not been widely accepted [34, 35]. High-dose ifosfamide could be used for these patients, especially when ifosfamide has not been applied previously to these same patients [36]. High-dose ifosfamide has been reported to be effective against recurrent disease, in a comparison with the standard dose [37, 38]. 

As for the treatment of lung metastases which appear after the initial diagnosis, surgical resection is usually considered. Radiotherapy in the lung after the resection of a metastatic lesion is not undertaken in most cases. In general, when a solitary metastatic lesion in the lung is detected more than 24 months after the initial diagnosis, surgical resection and close observation are chosen [39, 40], whereas in the case of patients with a single site of disease recurrence after a rather short relapse-free interval of around less than 24 months, systemic chemotherapy together with resection of the lesion is beneficial [7]. 

### New Treatment

In order to improve the survival of metastatic patients or patients classes as poor-responders following the standard protocol of a three-drug chemotherapy regimen using cispatin, doxorubicin and high-dose methotrexate, several protocols comprising intensive chemotherapy, such as high-dose or prolonged chemotherapy with/without peripheral blood stem cell rescue, have been considered, but the benefit of these therapies is not conclusive in long-term follow-up [3, 41, 42]. Improvement in the delivery efficacy of current agents may result in improved clinical results. Liposomal encapsulation of doxorubicin has been shown to be effective in delivering doxorubicin to patients, therefore providing the possibility that the resistance to doxorubicin could be overcome [43]. Furthermore, the efficacy of an aerosolized formulation of cisplatin has been investigated, especially for osteosarcoma patients [7].

A variety of agents has been investigated for the treatment of osteosarcoma in clinical trials. Studies with monoclonal antibodies, such as 8H9, heat-shock protein inhibitors, such as 17-N-allylamino-17-demethoxygeldanamycin [17-AG], and small molecule inhibitors, such as gefitinib and erlotinib, have been undertaken. Antifolate of methotrexate, trimetrexate, has been developed for the treatment of methotrexate-resistant osteosarcoma [44]. In addition, a new compound, PNU-159548, may have beneficial effects when combined with conventional agents [45]. Endostatin is an inhibitor of angiogenesis, and was administered in combination with liposome to experimental animals with osteosarcoma [46]. Liposomal muramyl tripeptide phosphatidylethanolamine is an activator of monocytes and macrophages and induces the secretion of different cytokines (Interleukin 1[IL 1], IL 6, tumor necrosis factor α [TNFα]), and it has been tested in clinical studies on osteosarcoma patients [47, 48]. Moreover, an immunomodulator of interferon for use as maintenance therapy has also been tested [49].

Identifying the genes and signal transduction pathways responsible for the development and malignant behavior in osteosarcoma will lead to the discovery of new drugs and therapy. It has been suggested that inhibition of the mammalian target of the rapamycin (mTOR) pathway may be effective in osteosarcoma [50, 51]. Inhibitors of receptor tyrosine kinases, including insulin-like growth factor 1 receptor (IGF-1R) and a platelet-derived growth factor receptor (PDGFR) have been investigated and developed for osteosarcoma treatment [52-54]. It has been reported that sustained-release sandostatin successfully performed the function of reducing serum IGF-1 levels [55]. Gene therapy is approaching readiness for clinical studies, and it now offers potentially promising results. The effectiveness of p53 gene therapy via a transferring-liposome-p53 complex administered in animal models has also been reported [56].

The receptor activator of nuclear factor-κβ, its ligand and the osteoprotegerin (RANK/RANKL/OPG) system is important in bone remodeling and metabolism [57, 58]. This RANK/RANKL/OPG system possibly plays a role in the pathogenesis of osteosarcoma [59, 60], and its interference may provide a new therapeutic strategy. Bisphosphonates inhibit osteoclast action and the resorption of bone, and it has been demonstrated that bisphosphonates inhibit tumor growth *in vitro. *Moreover, the effect of bisphosphonates combined with the standard protocol of cisplatin, doxorubicin and high-dose methotrexate has been investigated for osteosarcoma [61-63]. Peroxisome proliferator activated receptor (PPAR)γ/retinoid X receptor (PPARγ/RXR) agonists may induce differentiation in osteosarcoma, despite critical blocks to the normal bone morphogenetic protein (BMP)-regulated pathways. Differentiation therapy in osteosarcoma could be used as a new strategy [64].

## CONCLUSION

Osteosarcoma is a highly aggressive tumor with a high metastasizing potential. There is no established second-line chemotherapy, and accordingly, improvements in chemotherapy are desperately needed. Using microarray analysis in osteosarcoma, it has been demonstrated that a specific expression signature can differentiate between chemoresponsive and chemoresistant tumors [65]. Understanding the molecular mechanism of osteosarcoma development and malignant behavior will undoubtedly lead to the discovery of new chemotherapy agents.
